# An Act to Regulate the Practice of Medicine in the State of Georgia

**Published:** 1881-11

**Authors:** 


### Legislation.

### AN ACT

To regulate the practice of medicine in the State of Georgia.

SECTION 1. The General Assembly of the State of Georgia do enact, That no person shall practice medicine within this State unless he has been heretofore legally authorized so to do, or shall be hereafter authorized so to do, by a diploma from an incorporated medical college, medical school or university, and by compliance with subsequent sections of this Act.

SEC. 2. Be it further enacted, That, for the purposes of this Act, the words "practice medicine" shall mean to suggest, recommend, prescribe, or direct, for the use of any person, any drug, medicine, appliance, apparatus, or other agency, whether material or not material, for the cure, relief or palliation of any ailment or disease of the mind or body, or for the cure or relief of any wound, fracture, or other bodily injury, or any deformity, after having received, or with the intent of receiving therefor, either directly or indirectly, any bonus, gift or compensation.

SEC. 3 Be it further enacted, That every person now lawfully engaged in the practice of medicine within this State shall, on or before the first day of December, eighteen hundred and eighty-one, and every person hereafter duly qualified to practice medicine, shall, before commencing to practice, register in the office of the clerk of the superior court of the county wherein he resides and is practicing, or intends to commence the practice of medicine, in a book to be kept for the purpose by said clerk, his name, residence and place of birth, together with his authority for practicing medicine, as prescribed in this Act. The person so registering shall subscribe or verify, by oath or affirmation, before a person duly qualified to administer oaths under the laws of this State, an affidavit containing such facts, and whether such authority is by diploma or license, and the date of the same and by whom granted, which shall be exhibited to the county clerk before the applicant shall be allowed to register, and which, if wilfully false, shall subject the affiant to conviction and punishment for false swearing; the county clerk to receive a fee of fifty cents for each registration, to be paid by the person so registering.

SEC. 4. Be it further enacted, That every registered physician in this State who may change his residence from one county into another county within this State, shall register in the clerk's office of the county to which he removes and wherein he intends to reside and to practice medicine, as provided in section three (3) of this Act.

SEC. 5. Be it further enacted, That any person who violates either of the four preceding sections of this Act, or who shall practice or offer to practice medicine without lawful authority or under cover of a diploma or license illegally obtained, shall be deemed guilty of a misdemeanor, and, on conviction, shall be punished by a fine of not less than one hundred dollars or more than five hundred dollars, or by imprisonment for not less than thirty or more than ninety days, or both. The fine, when collected, shall be paid, the one-half to the person, persons or corporation making the complaint, the other half into the county treasury.

SEC. 6. Be it further enacted, That nothing in this Act shall apply to commissioned medical officers of the United States army or navy, or to the United States Marine Hospital service, or to legally qualified dentists in the practice of their profession, or to any woman practicing only midwifery.

SEC. 7. Be it further enacted, That all provisions of law providing for the organization, qualification and duties of any and all Boards of Physicians, or of any school whatever, be, and the same are hereby repealed; and there shall henceforth exist in this State no Board of Physicians, but the only requisite qualifications of practitioners of medicine shall be those hereinbefore set forth.

SEC. 8. Repeals conflicting laws.

Approved, September 28th, 1881.

